# Chronic DDE Exposure Modifies Mitochondrial Respiration during Differentiation of Human Adipose-Derived Mesenchymal Stem Cells into Mature Adipocytes

**DOI:** 10.3390/biom11081068

**Published:** 2021-07-21

**Authors:** Iva Kladnicka, Miroslava Cedikova, Jan Jedlicka, Michaela Kohoutova, Ludek Muller, Iveta Plavinova, Michaela Kripnerova, Monika Bludovska, Jitka Kuncova, Dana Mullerova

**Affiliations:** 1 Department of Public Health and Preventive Medicine, Faculty of Medicine in Pilsen, Charles University, 301 00 Pilsen, Czech Republic; Iveta.Plavinova@lfp.cuni.cz (I.P.); Monika.Bludovska@lfp.cuni.cz (M.B.); Dana.Mullerova@lfp.cuni.cz (D.M.); 2 NTIS, European Center of Excellence New Technologies for the Information Society, University of West Bohemia, 301 00 Pilsen, Czech Republic; muller@kky.zcu.cz; 3 Department of Physiology, Faculty of Medicine in Pilsen, Charles University, 301 00 Pilsen, Czech Republic; Miroslava.Cedikova@lfp.cuni.cz (M.C.); Jan.Jedlicka@lfp.cuni.cz (J.J.); Michaela.Kohoutova@lfp.cuni.cz (M.K.); Jitka.Kuncova@lfp.cuni.cz (J.K.); 4 Biomedical Centre, Faculty of Medicine in Pilsen, Charles University, 301 00 Pilsen, Czech Republic; 5 Department of Biology, Faculty of Medicine in Pilsen, Charles University, 301 00 Pilsen, Czech Republic; Michaela.Kripnerova@lfp.cuni.cz; 6 Department of Pharmacology and Toxicology, Faculty of Medicine in Pilsen, Charles University, 301 00 Pilsen, Czech Republic

**Keywords:** human adipose-derived mesenchymal stem cells, adipogenesis, p,p´-DDE, mitochondrial respiration

## Abstract

The contribution of environmental pollutants to the obesity pandemic is still not yet fully recognized. Elucidating possible cellular and molecular mechanisms of their effects is of high importance. Our study aimed to evaluate the effect of chronic, 21-day-long, 2,2-bis (4-chlorophenyl)-1,1-dichlorethylenedichlorodiphenyldichloroethylene (p,p´-DDE) exposure of human adipose-derived mesenchymal stem cells committed to adipogenesis on mitochondrial oxygen consumption on days 4, 10, and 21. In addition, the mitochondrial membrane potential (MMP), the quality of the mitochondrial network, and lipid accumulation in maturing cells were evaluated. Compared to control differentiating adipocytes, exposure to p,p´-DDE at 1 μM concentration significantly increased basal (routine) mitochondrial respiration, ATP-linked oxygen consumption and MMP of intact cells on day 21 of adipogenesis. In contrast, higher pollutant concentration seemed to slow down the gradual increase in ATP-linked oxygen consumption typical for normal adipogenesis. Organochlorine p,p´-DDE did not alter citrate synthase activity. In conclusion, in vitro 1 μM p,p´-DDE corresponding to human exposure is able to increase the mitochondrial respiration per individual mitochondrion at the end of adipocyte maturation. Our data reveal that long-lasting exposure to p,p´-DDE could interfere with the metabolic programming of mature adipocytes.

## 1. Introduction

Adipose tissue is a complex heterogeneous and highly dynamic organ performing many functions. It contributes to the control of energy metabolism of the whole organism. The specific function of adipose tissue is to provide mature adipocytes, i.e., cells that are able to store energy in lipid droplets in the form of triglycerides and release it in the chemical or thermal form according to the body requirements [1]. Adipogenesis, the process during which the mature adipocytes differentiate from their precursors, mesenchymal stem cells, is necessary for the dynamic renewal and optimal function of adipose tissue. It is supposed that the increase in adipocyte number is triggered by signaling factors that induce the commitment of pluripotent mesenchymal stem cells residing in the vascular stroma to the adipocyte lineage. When committed, preadipocytes are subjected to mitotic clonal expansion undergoing two or three cell divisions and then they gradually acquire typical adipocyte metabolic and morphological characteristic in the process of differentiation [2]. In the course of adipogenesis, mitochondrial oxygen consumption progressively increases. During the first days of adipogenesis, mitochondrial oxygen consumption is needed for the transition of differentiating cells from glycolytic to oxidative metabolism and the clonal expansion of preadipocytes, and then more energy is needed to acquire the typical metabolic phenotype of mature adipocyte [3].

Mature adipocytes make up only 20–30% of the total number of cells in adipose tissue. The remaining cells are made up of stromal vascular fraction and belong to the immune, epithelial, vascular, and stromal cells [4]. Besides storage and distribution of energy, adipose tissue contributes to the regulation of systemic energy metabolism by means of adipokines secretion such as adiponectin, leptin, resistin, interleukin-6 (IL-6), and tumor necrosis factor α. The secretion of adipokines enables autocrine, paracrine, endocrine, and cross talk communication with other organs [5]. Physiological production of adipokines requires intact cellular machinery of mature adipocyte, in particular mitochondrial respiration and balance between lipogenesis and lipolysis. Dysfunctional secretion of adipokines and free fatty acids induces an inflammatory response that is supposed to be the basis of peripheral insulin resistance [6]. These processes are linked to the specific distribution and accumulation of visceral fat, its morphological and inflammatory restructure, which are the main causes of metabolic complications, like diabetes mellitus type 2 and increased cardiovascular risk, even in a population with a normal body weight [7,8].

According to the last definition of the European Commission, obesity is depicted as a chronic relapsing disease, which in turn acts as a gateway to a range of other non-communicable diseases, such as diabetes, cardiovascular diseases, and cancer. Over the last forty years, the prevalence of obesity is increasing worldwide, achieving pandemic levels [9]. Many epidemiological and experimental studies suggest that obesity and associated dysfunction of adipose tissue might be a consequence of several interconnected causes such as genetic, environmental, and social factors [10,11].

The obesogenic environment is crucial in this trend. One of the possible characteristics of the obesogenic environment is chronic exposure to environmental contaminants, especially organochlorines, like pesticide 1,1,1-trichloro-2,2,-bis[p-chlorophenyl]ethane (DDT) [12]. It has been documented that DDT and its metabolite 2,2-bis (4-chlorophenyl)-1,1-dichlorethylenedichlorodiphenyldichloroethylene (p,p´-DDE) are associated with the increased risk of obesity and type 2 diabetes mellitus, and therefore they are called “metabolic disruptors” or “obesogens” [13,14,15,16]. Although production of DDT was banned in the late 1970s, its metabolites, especially p,p´-DDE can still be detected in human serum and samples rich in fat, such as breast milk [17,18].

DDE is accumulated and stored in lipophilic tissues, especially in adipose tissue. The variability of the stored amount of DDE in adipose tissue can range over several orders of magnitude, depends on dietary exposure and on individual disposition to store these substances [19,20]. For example, in the Smeds and Saukko study, DDE concentration levels in human adipose tissue ranged from 3.5 to 3229 ng/g lipids [13]. That serum concentration levels in men may exceed 3000 ng/g lipids was also confirmed [14]. These high values of serum concentration, based on lipid weight conversion correspond to 0.1 μM DDE. Concentration in the adipose tissue could be even one order higher.

The obesogenic action of these compounds disrupts homeostatic control over energy balance leading to overabundance in the metabolic pathways involved in energy storage over those that are responsible for metabolic energy expenditure flow [21]. Increasing evidence suggests that mitochondria might be a key player in the development of obesity. Multiple experimental studies demonstrated the inhibitory effects of organochlorines on mitochondrial oxygen consumption in the liver, brain, or skeletal muscle [22,23]. The impact of long-lasting DDE exposure on mitochondrial respiration of differentiating adipocytes has not been studied yet [24].

In our study, we employed human adipose-derived mesenchymal stem cells (hADMSCs) committed to adipogenesis. The effects of p,p´-DDE in concentrations 1 μM and 10 μM on differentiating adipocyte mitochondrial oxygen consumption, citrate synthase activity, and mitochondrial membrane potential were evaluated on days 4, 10, and 21 of adipogenesis. In addition, lipid accumulation and mitochondrial quality were assessed to verify the phenotype of differentiating cells.

## 2. Materials and Methods

### 2.1. Cell Culture and Differentiation In Vitro

Human adipose-derived mesenchymal stem cells were isolated from a female donors’ subcutaneous adipose tissue, purchased from Thermo Fisher (Thermo Fisher Scientific, Carlsbad, CA, USA) and cultivated. The cells were seeded at 1 × 10^5^ cells/cm^2^ and cultured in Petri dishes (TPP Techno Plastic Products, Trasadingen, Switzerland) in commercially available culture medium MesenPRO RS™. The medium was supplemented with MesenPRO RS™ Growth Supplement with reduced serum level (2%), 1% l-glutamine, and 1% gentamicin (all Thermo Fisher Scientific, Carlsbad, CA, USA).

**Figure 1:**
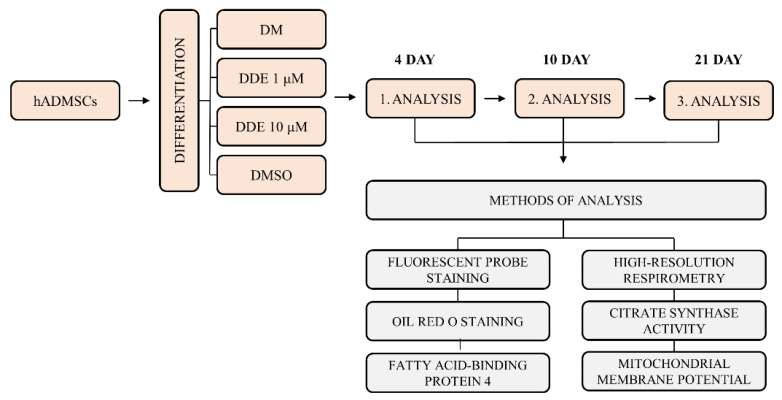
Summary scheme of the experiment.DM: pure differentiation medium; DDE 1 μM: differentiation medium with added p,p´-DDE, which was dissolved in dimethyl sulfoxide (DMSO); DDE 10 μM: differentiation medium with added p,p´-DDE, which was dissolved in DMSO; DMSO: differentiation medium (DM) with DMSO only.

After reaching 80% confluence, the culture medium was changed to four different differentiation media: (A) pure differentiation medium (DM); (B) differentiation medium with dimethyl sulfoxide (DMSO); (C) differentiation medium with DMSO and p,p´-DDE, in final concentration 1 μM (DDE 1 μM); (D) differentiation medium with DMSO and p,p´-DDE in final concentration 10 μM (DDE 10 μM). The pure differentiation medium contained StemPro^®^ Adipogenesis Differentiation Basal Medium with StemPro^®^ Adipogenesis Supplement and 1% gentamicin (all Thermo Fisher Scientific, Carlsbad, CA, USA). DDE concentrations 1 μM and 10 μM were chosen because of their most common use in in vitro studies. DMSO, which is commonly used as a solvent of lipophilic compounds for in vitro cell experiments, is generally considered nontoxic up to 0.05% concentration (*v*/*v*) [18]; however, some studies suggest that this solvent can even promote proliferation at least in some cell lines [25]. On that account, we first compared the differentiation process under DM alone with the differentiation medium with DMSO in concentration used in our experiments, i.e., 0.019%. We have found that the addition of DMSO affected the process of differentiation, since the cell count per well measured using NucBlue^TM^ Live ReadyProbes^TM^ Reagent (Thermo Fisher Scientific, Carlsbad, CA, USA) as described below displayed a slightly different pattern in the course of adipogenesis. Thus, all our results acquired from DDE-exposed cell cultures were compared separately to the cells differentiating in the same medium with or without DMSO. A summary scheme of the experiment is shown in Figure 1.

The medium was changed every 3 days up to a total incubation time of 21 days. The cells were maintained and cultured into differentiated adipocytes under 5% CO_2_ atmosphere at 37 °C [26]. The independent experimental number for each method was 12 except in high-resolution respirometry. With the aim to prove that the experimental system was running as expected, we monitored the process of adipogenesis by Oil Red O staining and fluorescent probe staining methods.

### 2.2. High-Resolution Respirometry

**Figure 2:**
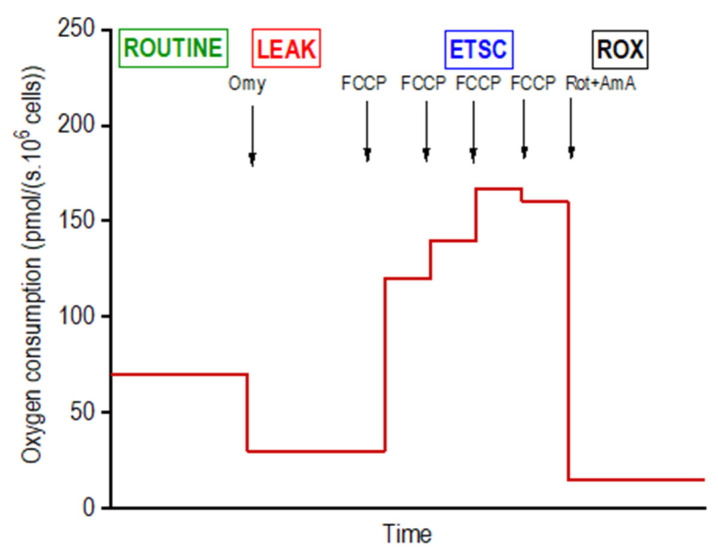
Design of a titration protocol for measuring the mitochondrial oxygen consumption in intact differentiating adipocytes. For the concentrations of inhibitors and uncoupler utilized and definition of respiratory states, see Methods.

Mitochondrial respiration of intact adipocytes on days 4 (*n* = 6 per group; i.e., DM, DMSO, DDE 1 µM, DDE 10 µM), 10 (*n* = 6) and 21 (*n* = 6) of differentiation was analyzed in the StemPro^®^ Adipogenesis Differentiation medium using high-resolution respirometry in a 2-chamber oxygraph O2k (Oroboros Instruments, Innsbruck, Austria). The negative time derivative of the oxygen concentration in the chamber was calculated online to determine oxygen consumption (DatLab software, version 7.3.0.3, Oroboros, Austria). The cells were applied into the open precalibrated oxygraph chambers, stirred at 350 rpm and a sample of 15 µL of the cell suspension was aspirated to count the cells in the Bürker hemocytometer. After closing the oxygraph chambers, a substrate-uncoupler-inhibitor titration (SUIT) protocol for intact cells was used to determine the standard respiratory states: ROUTINE (ROUT; R)—oxygen consumption at the physiological coupled state; LEAK (L; injection of 2.5 µmol/L oligomycin)—non-phosphorylating resting state of respiration to compensate for the proton leak when ATP synthase is not active; the electron-transfer-system capacity (ETSC; E) was assessed after titrations of trifluorocarbonylcyanide phenylhydrazone (FCCP; 0.05 µmol/L titration steps) to reach maximal oxygen consumption in the noncoupled state; and ROX, residual oxygen consumption indicating oxidative side reactions remaining after inhibition of the electron transfer pathway (0.5 μmol/L rotenone and 2.5 µmol/L antimycin A) [21]. Oxygen consumption was expressed in pmoL O_2_/(s · 10^6^ cells) and corrected to ROX and instrumental background. The representative scheme of the experiment on intact cells is shown in Figure 2.

In addition, the following control parameters were calculated: L/E coupling control ratio as an index of uncoupling; R/E control ratio showing how close ROUT operates to ETSC; E-R reserve capacity reflecting the difference between noncoupled and coupled respiration; R-L or net routine capacity related to the cellular ATP production.

### 2.3. Citrate Synthase Activity

The determination of citrate synthase activity was used to estimate mitochondrial content in the samples from each oxygraph chamber. The assay medium for citrate synthase activity consisted of 0.1 mmol/L 5,5-dithio-bis- (2-nitrobenzoic) acid, 0.25% triton-X, 0.5 mmol/L oxaloacetate, 0.31 mmol/L acetyl coenzyme A, 5 µmol/L EDTA, 5 mmol/L triethanolamine hydrochloride, and 0.1 mol/L tris-HCl, pH 8.1. One hundred microliters of the mixed and homogenized chamber content were added to 900 µL of the medium. The rate of absorbance change was measured spectrophotometrically at 412 nm and 30 °C over 200 s.

### 2.4. Mitochondrial Membrane Potential

The mitochondrial membrane potential (MMP) was measured using the JC-1 Mitochondrial Membrane Potential Assay Kit (Mitosciences, Abcam, Cambridge, UK). The cells were seeded at 1 × 10^4^ cells on a dark 96-well plate and cultured as explained earlier. The MMP was evaluated on days 4 (*n* = 12), 10 (*n* = 12), and 21 (*n* = 12) of differentiation. The cells were washed once with phosphate buffered saline (PBS) and incubated with JC-1 dye (1 μM) at 37 °C for 10 min. Then the cells were rinsed twice with PBS and were analyzed on a fluorescence spectrophotometer (Synergy HT, BioTek, Winooski, VT, USA) at excitation 475 nm and emission 530/590 nm. The red/green fluorescence intensity ratio was determined to evaluate MMP.

### 2.5. Oil Red O, Fluorescent Probe Staining, and Fatty Acid-Binding Protein 4

All these methods have been used to prove that the experimental system was performing as expected.

#### 2.5.1. Oil Red O Staining

Oil Red O is a fat-soluble dye that stains neutral triglycerides and lipids [27]. Intracellular triglyceride droplets of hADMSCs were stained with Oil Red O solution on day 4, 10, and 21. The sample preparation procedure was as follows: medium was removed, each well was rinsed twice in PBS, and fixed in 4% formaldehyde for 1 h at room temperature. The cells were then rinsed twice with distilled water and stained with Oil Red O solution (0.5 g of Oil Red O powder dissolved in 100 mL of isopropanol). This solution was blended with distilled water in the ratio 3:2. Cells were incubated with this solution for 15 min at room temperature, then washed twice with distilled water and visualized using the Olympus CX41 microscope (Olympus, Tokyo, Japan) connected to a digital camera.

**Figure 3:**
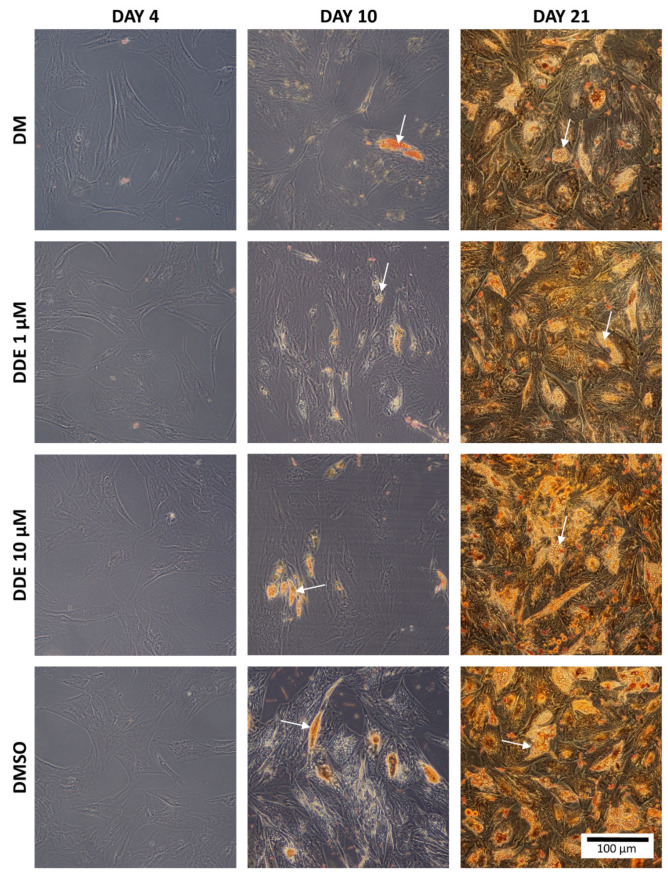
The comparison of fat in each environment. The arrows show examples of fat droplets. (Oil Red O staining).

Figure 3 shows the intracellular fat droplets during differentiation in all four types of the media.

#### 2.5.2. Fluorescent Probe Staining

**Figure 4:**
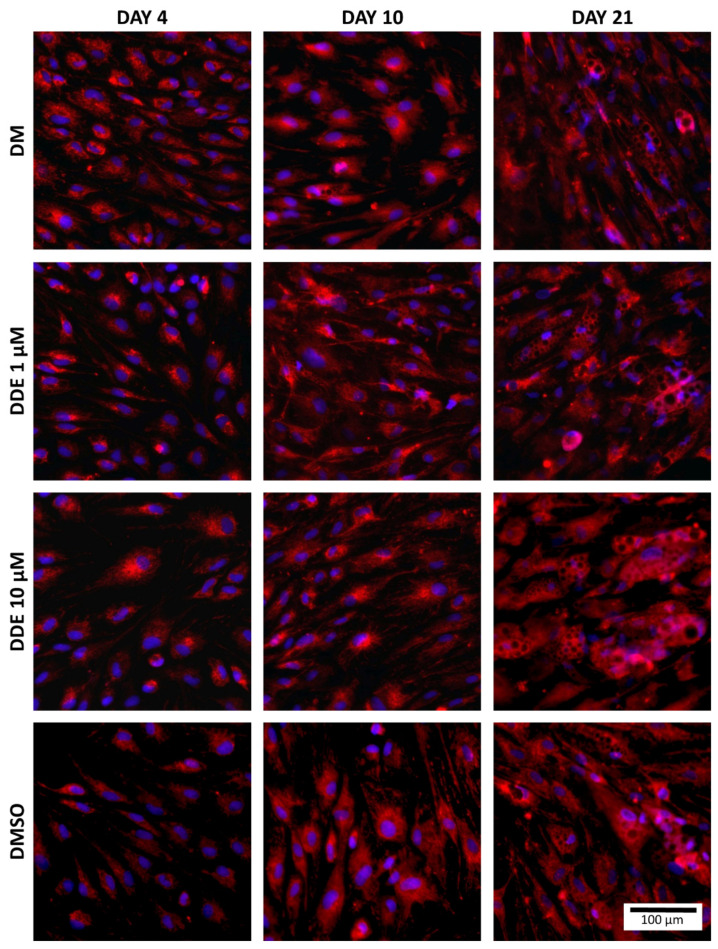
Staining of mitochondria (red) and nuclei (blue) in each environment (fluorescent probes, MitoTracker^TM^ Red CMXRos and NucBlue^TM^ Live ReadyProbes Reagent were used to visualize the mitochondria and nucleus of cells).

Fluorescent probes, MitoTracker^TM^ Red CMXRos and NucBlue^TM^ Live ReadyProbes Reagent (both Molecular Probes, Eugene, OR, USA) were used to visualize the mitochondria and nuclei of cells. MitoTracker^TM^ passively passes into mitochondria and accumulates there. NucBlue^TM^ Reagent contains Hoechst 33342 (2′-[4-ethoxyphenyl]-5-[4-methyl-1-piperazinyl]-2,5′-bi-1H-benzimidazole) which emits a blue fluorescence when bound to DNA (Figure 4).

The cells were first stripped of differentiation medium, which was replaced with special medium for live cell imaging—Live Cell Imaging Solution (Molecular Probes, Eugene, OR, USA). Subsequently, Mitotracker^TM^ was added to the medium (final concentration 100 nM) and two drops per millilitre of NucBlue^TM^ were added too. Cells were incubated in the dark for 30 min and then visualized by the Hamamatsu Orca-ER camera mounted on the Olympus IX 81 inverted microscope at 200× magnification (Olympus, Tokyo, Japan).

#### 2.5.3. Fatty Acid-Binding Protein 4

Fatty acid-binding protein (FABP4) is highly expressed in adipocytes and consists of about 1% of all soluble proteins in adipose tissue [28]. On day 21, the adipogenic marker FABP4 was measured to confirm the presence of adipocytes.

Cells were washed in phosphate buffered saline (PBS), fixed for 60 min in 4% formaldehyde with PBS at room temperature, and permeabilized in PBS containing 0.3% Triton X-100 for 15 min followed by blocking in PBS with 1% bovine serum albumin (BSA) and 10% normal donkey serum at room temperature for 60 min. After blocking, cells were incubated with anti-mFABP4 antibody (anti-mouse Fatty Acids Binding Protein 4, R&D Systems, Inc., Minneapolis, MN, USA) working solution (PBS containing 0.03% Triton X-100, 1% BSA, 10% normal donkey serum and anti-mFABP4 in final concentration 10 µg/mL) overnight at 2–8 °C. After three 5-min rinses in PBS with 1% BSA, cells were incubated for 1 h in NL557-conjugated anti-goat secondary antibody (R&D Systems, Inc., Minneapolis, MN, USA) diluted 1:200 in 1% BSA in PBS in the dark for 60 min at room temperature. The coverslips were washed, placed on microscope slides with a mounting medium (ProLong Gold Antifade Mountant with DAPI, Molecular Probes, Eugene, OR, USA) and visualized using the Hamamatsu Orca-ER camera mounted on the Olympus IX 81 inverted microscope at 200× magnification (Olympus, Tokyo, Japan).

### 2.6. Data Analysis and Statistics

The data were processed with the use of the statistical software MATLAB Statistics Toolbox (MathWorks Inc., Natick, MA, USA) and OriginPro 2017 (OriginLab Corp., Northampton, MA, USA). After testing for normality of distribution (Shapiro Wilk test), normally distributed data were analyzed using two-way ANOVA followed by the Tukey post hoc test. Non-normally distributed data were processed by log-transformation and then analyzed accordingly. In addition, the differences between groups that required transformation or data that could not reach normal distribution were analyzed using the Wilcoxon rank sum and Friedman statistical tests. *p*-values ≤ 0.05 were considered statistically significant.

## 3. Results

### 3.1. High-Resolution Respirometry

**Figure 5:**
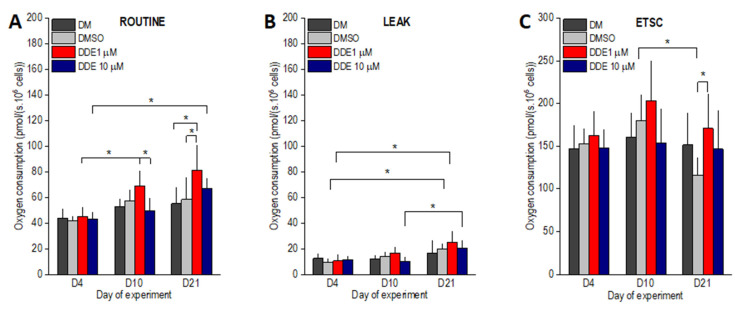
(**A**–**C**) ROUTINE, LEAK and uncoupled (ETSC) oxygen consumptions of adipocytes differentiating in media containing no additive (DM) or DMSO, DMSO and 1 µM p,p’-DDE, and DMSO and 10 µM p,p’-DDE on days 4, 10, and 21 of the experiment. Significant differences * *p* < 0.05 (Two-way ANOVA followed by a post-hoc Tukey test).

As expected, in the control samples (DM and DMSO), the routine respiration increased between days 4 and 10 of the experiment by ~30%. However, the differences did not reach statistical significance (Figure 5A). In contrast, routine oxygen consumption observed in DDE 1 µM samples on day 10 of adipogenesis was ~50% higher than on day 4 (*p* < 0.0001) and became significantly different also when compared to DDE 10 µM cells. DDE 10 µM adipocytes did not display any increase in the state ROUT between days 4 and 10 of adipogenesis. In cells differentiating in the control media only (DM and DMSO), routine respiration remained nearly the same on day 21. DDE 10 µM cells had higher routine oxygen consumption on day 21 compared to day 4. In adipocytes differentiating in the presence of 1 µM DDE, routine oxygen consumption further increased on day 21 of the experiment and became significantly different compared to both DMSO and DM controls (Figure 5A). The LEAK state, i.e., oxygen consumption after ATP synthesis inhibition by oligomycin gradually increased from day 4 to 21 in the control (DMSO) and DDE 1 µM samples reaching significant difference between days 4 and 21 of the experiment. In DDE 10 µM adipocytes, the leak respiration was very similar on days 4 and 10 (11.6 ± 2.9 and 10.1 ± 3.2 pmol/s · 10^6^ cells, respectively) and then increased reaching significant difference between days 10 and 21. No significant difference between the treatments was noted on days 4, 10, or 21 of the experiment (Figure 5B). ETSC did not differ between days 4 and 10 and then tended to decline resulting in a significant difference between the DMSO control and DDE 1 µM adipocytes on day 21 of the experiment. In addition, the ETSC state of DMSO control cells was significantly lower on day 21 compared to day 10 (Figure 5C).

**Figure 6:**
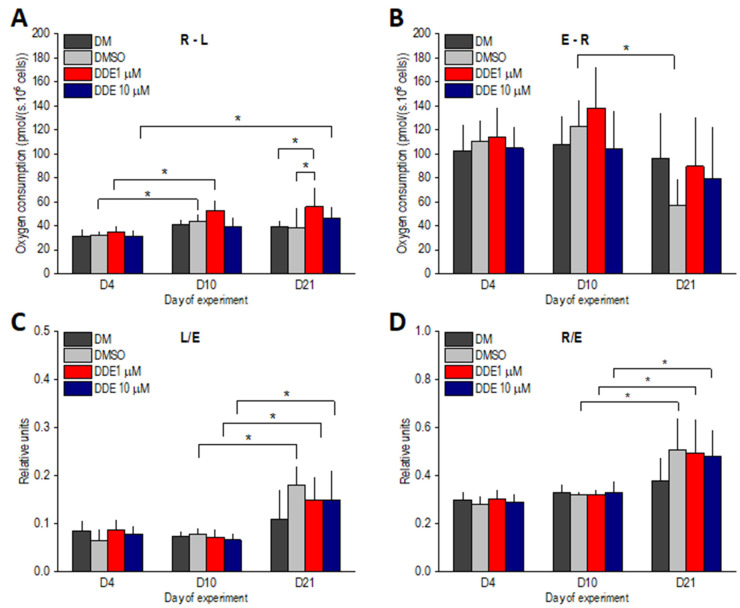
(**A**,**B**) ATP-related oxygen consumption (R-L) and reserve ETS capacity (E-R) in adipocytes differentiating in media containing no additive (DM) or DMSO, DMSO and 1 µM p,p’-DDE, and DMSO and 10 µM p,p’-DDE on days 4, 10, and 21 of the experiment. (**C**,**D**) Flux control ratios L/E and R/E. Significant differences * *p* < 0.05 (Friedman and Wilcoxon rank sum tests).

ATP-linked oxygen consumption, i.e., the R-L state, slightly increased between days 4 and 10 in all groups of differentiating adipocytes and then remained stable. Significant differences between days 4 and 10 of adipogenesis were reached only in DDE 1 µM and DMSO-control samples. On day 21 of the experiment, R-L was significantly higher in DDE 1 µM compared to DM and DMSO control groups. Reserve respiratory capacity, i.e., the E-R state, decreased between days 10 and 21; however, this decline was significant only in DMSO adipocytes. The L/E coupling control ratio significantly increased in all DMSO-containing samples on day 21, suggesting that the extent of uncoupling was similar in controls and DDE-treated adipocytes. Similarly, the R/E control ratio increased to a comparable extent in all groups of adipocytes on day 21 of the experiment, documenting that in the later stages of differentiation, routine respiration operates closer to ETSC (Figure 6).

Citrate synthase activity ranged between 10.61 ± 5.26 to 18.32 ± 4.74 mIU/10^6^ cells in DMSO control cells on day 21 and DDE1 adipocytes on day 10, respectively. No significant difference was noted between individual groups on any day of the experiment nor between adipocytes after the same intervention in the course of the experiment. Data not shown.

### 3.2. Mitochondrial Membrane Potential

**Figure 7:**
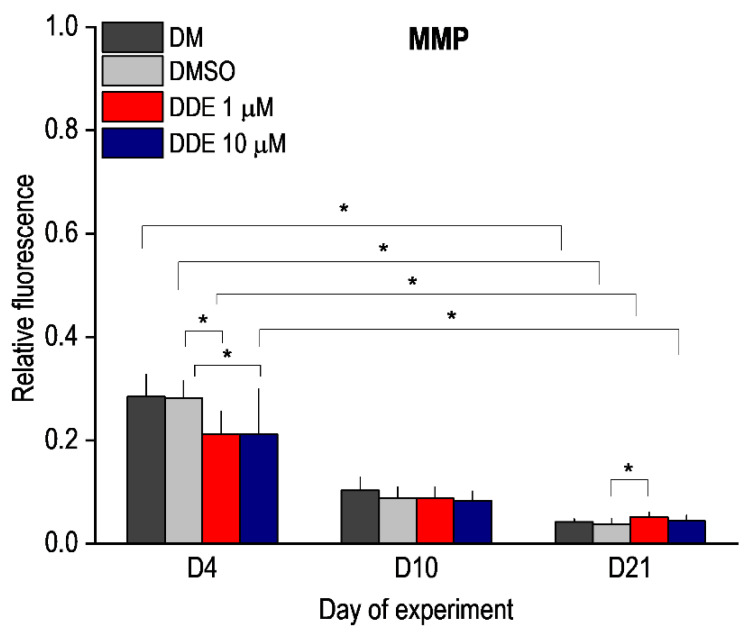
Mitochondrial membrane potential (MMP) during adipocyte differentiation in media containing no additive (DM) or DMSO, DMSO, and 1 µM p,p’-DDE, and DMSO and 10 µM p,p’-DDE on days 4, 10, and 21 of the experiment. Significant differences * *p* < 0.05 (Friedman and Wilcoxon rank sum tests). On the fourth day of differentiation, a significant decrease in MMP was observed in DDE-affected cells at both 1 μM and 10 μM concentrations compared to medium containing DMSO alone. In cells affected with 1 μM DDE, there was a significant increase in MMP on day 21 of differentiation compared to control cells exposed to DMSO alone.

MMP is one of the key parameters of mitochondrial function and serves as an indicator of cell health—regulation of ATP synthesis, ROS production, calcium sequestration, etc. In healthy cells, JC-1 dye enters the mitochondria and forms red aggregates. Mitochondria in cells with low mitochondrial potential do not form aggregates and remain in monomeric form with green fluorescence. We compared JC1 staining in unaffected adipocyte differentiation and in differentiation under the influence of DMSO and DMSO with DDE. In all cases, we observed a gradual decrease in the red/green ratio during differentiation, which corresponds to previous findings [3,29]. On the fourth day of differentiation, a significant decrease in MMP was observed in DDE-affected cells at both 1 μM and 10 μM concentrations compared to medium containing DMSO alone. In cells affected with 1 μM DDE, there was a significant increase in red/green ratio on day 21 of differentiation compared to cells exposed to DMSO alone (Figure 7).

## 4. Discussion

In our study, we monitored chronic DDE exposure at two different concentrations to a developing adipocytes differentiating from hADMSCs. We found that the long-lasting treatment with DDE in a concentration of 1 μM in contrast to 10 μM resulted in statistically significant deviations in mitochondrial respiration compared to control cells. The characteristic pattern of changes in mitochondrial respiration during adipogenesis was not impaired by lower DDE concentration showing a typical gradual increase in the routine and leak respirations of maturing adipocytes [3]. However, on day 21 of the experiment—i.e., in adipocytes regarded as fully differentiated—higher mitochondrial membrane potential, higher resting mitochondrial oxygen consumption (routine respiration), and higher ATP-related respiration (R-L) were observed in DDE 1 μM samples compared to relevant controls. Our results suggest that lower concentrations of the pollutant during chronic exposure may more significantly modulate physiological processes, in this case probably through an endocrine disrupting effect. Such a finding might seem surprising since the majority of studies dealing with the impact of organochlorines on mitochondrial functions reported impaired oxygen consumption of the tissues studied [27,28,29,30,31]; reviewed in [12]. However, it should be noted that the concentrations of DDT and DDE used in these studies were relatively high and their effects were analyzed mainly on the liver and muscle mitochondria after a single dose exposure. The design of our experiment differs in the chronicity of exposure, lower concentrations of the pollutant used, the continuous action of DDE on the whole process of adipocyte differentiation until maturation, and the type of affected cells.

Appropriate mitochondrial function in general and oxidative phosphorylation in particular is essential for ATP production and the whole-body energy homeostasis. Mitochondrial dysfunction has been implicated in the development of many pathological conditions associated with obesity as a result of imbalance between food intake and energy expenditure, such as type 2 diabetes mellitus. Nevertheless, mitochondrial dysfunction does not necessarily mean that mitochondrial oxygen consumption and ATP production should be decreased in all tissues involved in the regulation of energy homeostasis. Tissue-specific control of mitochondrial respiration was demonstrated in obese diabetic mice displaying impaired mitochondrial respiration in the liver and oxidative skeletal muscle, but enhanced oxygen consumption in glycolytic skeletal muscle [32]. Another study reported increased mitochondrial oxygen consumption in the brown fat of obese mice [33]. In addition, adipocyte-specific decrease in oxidative phosphorylation affected systemic energy homeostasis and protected against the development of obesity and insulin resistance in mice fed a high-fat diet [34]. In the study conducted by Böhm et al. (2020), enhanced mitochondrial respiration of adipocytes isolated from obese insulin-resistant donors was attributed to adaptation of the cellular metabolism to the increased amount of fuels associated with insulin resistance [35].

In our study, the supply of metabolic substrates was the same in all experimental groups; however, increased ATP demand in DDE-treated adipocytes could be related to the promoting effect of DDE and similar pollutants on the de novo synthesis of lipids and their accumulation [36]. The negative effects of DDT/DDE on thermogenic proteins expression and substrate transport/utilization in adipocytes were also documented, which could lead to an increased ATP need to compensate for compromised fuel transport or heat dissipation [37]. A recent study suggests that impaired thermogenesis in DDT-affected adipocytes could be caused by targeting mechanisms upstream of adipose tissue without the necessity to compromise the expression of uncoupling proteins [38]. In this study, the leak respiration needed to compensate for proton leak, electron slip, and cation cycling increased in the course of adipocyte differentiation in all groups to a similar extent, without reaching any significant difference between DDE-treated and control groups on day 21.

The excess E-R capacity (respiratory reserve) was the only parameter compromised by the long-lasting exposure to DMSO (between days 10 and 21 of differentiation). Nevertheless, the same trend was observed in DDE-treated adipocytes on day 21 verifying our previous finding that in the later stages of differentiation, the routine respiration could be increased only on the account of the total electron-transporting capacity of mitochondria [3]. In addition, the ratio of routine to maximal respiration (R/E) was nearly the same in DMSO-control and DDE-treated adipocytes on day 21 of the experiment and in all these groups it reached significantly higher values than on day 10 of differentiation.

Another interesting finding of this study is the fact that at higher concentration, i.e., 10 μM, DDE seemed to have no effect, since on day 21, no significant differences were revealed between DDE 10 μM and control adipocytes. However, DDE 10 μM–exposed differentiating adipocytes displayed a later onset of changes in mitochondrial parameters associated with normal adipogenesis, i.e., an increase in routine respiration and ATP-linked oxygen consumption. Such “bidirectional” action of various pollutants on the cellular functions is not a new finding and makes the research into the putative effects of endocrine disruptors more complicated [39].

Thus, evaluating the impact of DDE on mitochondrial functions in human differentiating adipocytes, adipogenesis can be attacked in two ways: (i) at higher DDE concentrations by slowing down the differentiation process, (ii) at lower chronic DDE levels by increased mitochondrial respiration and ATP generation, which then could lead to disturbances in energy homeostasis.

Mitochondrial dysfunction of the adipocyte may then be involved in the pathogenesis of obesity-related metabolic diseases, such as diabetes mellitus, in particular if the supply of metabolic substrates is increased simultaneously. Overexposure of cells to saturated fatty acids, which is also associated with higher exposure to DDT and DDE from food of animal origin might aggravate mitochondrial dysfunction of other tissues and contribute to disturbances of the whole-body energy balance [40].

## 5. Study Limitations

This study addressed the impact of persistent organochlorine pollutant p,p’-DDE on mitochondrial respiration in a single cell model. However, the adipocytes differentiating from hADMSCs have only limited survival time and cannot provide complex insight into impaired regulation of the functions of adipose tissue exposed to potential obesogens for the whole life. In future studies, long-lasting exposure to the pollutant should be extended to in vivo models to verify the putative disrupting effect of the compound on energy homeostasis and to specify the mechanism by which it could contribute to obesity and related diseases.

## 6. Conclusions

Elucidating the cellular and molecular mechanisms of DDE obesogenic effects is critical to understand the putative causal relationship of DDE to obesity and its metabolic complications.

This study focused on the impact of DDE on the metabolic characteristic of hADMSCs in the whole course of differentiation (21 days). Its results show that subtle sequelae of DDE chronic action could be observed at the end of differentiation, i.e., after long-lasting exposure. Maturing adipocytes “adapted” to continuous supply of the pollutant in lower doses (1 µM), displayed higher basal mitochondrial respiration and ATP-linked oxygen consumption along with impaired uncoupling reflected in higher mitochondrial membrane potential that might interfere with efficient heat dissipation. A higher concentration of the pollutant slows down the differentiation process, as documented by the later onset of the increase in ATP-linked oxygen consumption.

In conclusion, the hADMSCs in vitro model of differentiation is suitable to study the impact of chronic DDE exposure on different features of adipogenesis. The long-lasting action of DDE seems to result in metabolic reprogramming of adipocytes that might contribute to the obesogenic effect of the pollutant studied.

## Data Availability

Data available on request due to privacy restrictions. The data presented in this study are available on request from the corresponding author. The data are not publicly available due to the co-ownership of multiple workplaces.
